# Dendrimers and Polyamino-Phenolic Ligands: Activity of New Molecules Against *Legionella pneumophila* Biofilms

**DOI:** 10.3389/fmicb.2016.00289

**Published:** 2016-03-09

**Authors:** Elisa Andreozzi, Federica Barbieri, Maria F. Ottaviani, Luca Giorgi, Francesca Bruscolini, Anita Manti, Michela Battistelli, Luigia Sabatini, Anna Pianetti

**Affiliations:** ^1^Department of Biomolecular Sciences, University of Urbino Carlo BoUrbino, Italy; ^2^Department of Pure and Applied Sciences, University of Urbino Carlo BoUrbino, Italy

**Keywords:** *Legionella pneumophila*, biofilm formation, biofilm removal, polyamino-phenolic ligands, dendrimers

## Abstract

Legionnaires’ disease is a potentially fatal pneumonia caused by *Legionella pneumophila*, an aquatic bacterium often found within the biofilm niche. In man-made water systems microbial biofilms increase the resistance of legionella to disinfection, posing a significant threat to public health. Disinfection methods currently used in water systems have been shown to be ineffective against legionella over the long-term, allowing recolonization by the biofilm-protected microorganisms. In this study, the anti-biofilm activity of previously fabricated polyamino-phenolic ligands and polyamidoamine dendrimers was investigated against legionella mono-species and multi-species biofilms formed by *L. pneumophila* in association with other bacteria that can be found in tap water (*Aeromonas hydrophila*, *Pseudomonas aeruginosa*, *Escherichia coli*, *Klebsiella pneumoniae*). Bacterial ability to form biofilms was verified using a crystal violet colorimetric assay and testing cell viability by real-time quantitative PCR and Plate Count assay. The concentration of the chemicals tested as anti-biofilm agents was chosen based on cytotoxicity assays: the highest non-cytotoxic chemical concentration was used for biofilm inhibition assays, with dendrimer concentration 10-fold higher than polyamino-phenolic ligands. While Macrophen and Double Macrophen were the most active substances among polyamino-phenolic ligands, dendrimers were overall twofold more effective than all other compounds with a reduction up to 85 and 73% of legionella and multi-species biofilms, respectively. Chemical interaction with matrix molecules is hypothesized, based on SEM images and considering the low or absent anti-microbial activity on planktonic bacteria showed by flow cytometry. These data suggest that the studied compounds, especially dendrimers, could be considered as novel molecules in the design of research projects aimed at the development of efficacious anti-biofilm disinfection treatments of water systems in order to minimize legionellosis outbreaks.

## Introduction

*Legionella* genus includes aerobic, motile, gram-negative bacteria that are the etiological agents of legionellosis. The clinical spectrum and severity of the infection ranges from a self-limited, acute flu-like illness, to an atypical severe form of pneumonia called Legionnaires’ disease, characterized by a high mortality rate of 10–15% (Bartram et al., 2007). The most pathogenic species is *Legionella pneumophila*, responsible for more than 90% of described clinical cases (Steinert et al., 2002; Bartram et al., 2007), and strains belonging to serogroup 1 (Sg1) the ones predominantly isolated (Doleans et al., 2004; Ng et al., 2009). The reason for the higher pathogenicity of *L. pneumophila* Sg1 is not completely understood; its clinical prevalence appears not to be correlated with predominance in the environment, where non-Sg1 strains show higher recovery percentages. The low non-Sg1 sensitivity of the commonly used diagnostic method could be a reason for Sg1 clinical prevalence, however, previous studies link it to an increased resistance to the alternative serum complement pathway, due to variations in the outer-membrane *O*-antigen segment of the lipopolysaccharide. Moreover, Sg1 strain Philadelphia was proven to induce bacteraemia and disseminate to other organs in mice, unlike non-Sg1 strains (Khan et al., 2013).

*Legionella* sp. show ubiquitous distribution, occurring in different natural aquatic environments and artificial water systems (Doleans et al., 2004). *Legionella* transmission mostly occurs via aerosols generated by several potential sources such as air conditioning and hot water systems, cooling towers, humidifiers and shower heads (Doleans et al., 2004; Osawa et al., 2014). *Legionella* virulence is due to its ability to infect and grow in macrophages and its environmental persistence largely depends on the ability to proliferate in protozoa, especially in amoebae of genera *Acanthamoeba* and *Hartmannella* (Doleans et al., 2004; Messi et al., 2013).

*Legionella* sp. can survive in natural and man-made water environments as planktonic cells or as surface-associated cells, embedded in an extracellular polysaccharide matrix, forming mixed microbial biofilms (Bartram et al., 2007; Declerck et al., 2009; Stewart et al., 2012). Biofilm formation allows nutrient distribution and sharing of genetic material, facilitates intercellular communications by signal molecules and supplies cell protection from environmental stresses (Watnick and Kolter, 2000) and from biocides or disinfectant treatments that would inactivate free-floating microorganisms (Sanli-Yurudu et al., 2007; Bridier et al., 2011). Biofilms positively influence the persistence of *Legionella* in water systems and they may enhance the virulence of the bacteria inside the host. Khweek et al. (2013) found that biofilm-derived legionellae were able to avoid the phagosome-lysosome fusion in murine macrophages and to proliferate more rapidly in host cells in contrast to planktonic bacteria. Moreover, the presence of protozoa, such as amoebae, in water systems increases bacterial resistance to antimicrobial agents, acting as hosts for legionella replication and protectors within the intracellular niche (Declerck et al., 2009; Lau and Ashbolt, 2009). Intra-amoeba grown *Legionella* sp. appear to be even more pathogenic than the free-living form as they were found to be more invasive in several human cell lines (Lau and Ashbolt, 2009).

In order to prevent legionellosis outbreaks, different disinfection methods (water heating, ultraviolet radiation, chlorination, monochloramine, ozonation, copper and silver ionization, peroxides) have been used to eliminate legionellae or at least control their growth in water supplies (Miyamoto et al., 2000; Levin, 2009; Jakubek et al., 2013). However, most of the physical and chemical treatments have been shown not to be effective over a long time, mainly because of their inability to penetrate the biofilm, which strengthens the microbial tolerance to biocides, thereby favoring bacterial regrowth after decontamination (Ditommaso et al., 2005; Iatta et al., 2013). Even though monochloramine was found to cause a significant decrease of *L. pneumophila* in a nuclear power plant cooling circuit, the presence of biofilm allowed the re-colonization of the system when the treatment was interrupted (Jakubek et al., 2013). Furthermore, the most clinically relevant strains, *L. pneumophila* Sg 1, were shown to be the most resistant serogroup to chlorination in several studies, as they might develop or better adapt to protective biofilms (Cooper and Hanlon, 2010; Iatta et al., 2013).

Removal of biofilms means elimination of one of the main sources of recontamination after water system treatment and could be helpful to obtain a better performance of the commonly used disinfection methods that are effective on planktonic microorganisms but not on the sessile counterpart. Discovery and implementation of new potential and effective disinfection strategies is a major but essential challenge to minimize *Legionella* presence and consequently reduce legionellosis risk to public health. This goal could be achieved testing new molecules that are active against bacteria resistant to traditional biocides, as referred by Tiwari et al. (2015), and also active in biofilm control. Some anti-biofilm techniques have been developed and proposed, combining mechanical with physical or chemical processes. It is also suggested that addition of enzymatic molecules contributes to biocide activity improvement through destruction of the polysaccharide matrix (de Carvalho, 2007). However, new studies are necessary to improve the efficiency of biofilm removal. In order to test compounds that could be thought as novel molecules in the development of new and not only temporary disinfection strategies, we investigated the ability of polyamidoamine (PAMAM) dendrimers and polyamino-phenolic ligands to interfere with biofilms.

PAMAM dendrimers are repetitively branched molecules typically symmetric around the central core, where the number of ramifications determines the number of generations. The surface of PAMAM dendrimers exhibit carboxy (half-generation, e.g., G0.5 dendrimer) or amino groups (full-generation, e.g., G2 dendrimer), whose number increases with the number of generations (Tomalia et al., 2012). Dendrimers have been studied so far to act as drugs, drug carriers, vehicle of biological materials and antimicrobial agents (Chen and Cooper, 2000; Ottaviani et al., 2000, 2004; Klajnert et al., 2006; Tülü and Ertürk, 2012). Dendrimers could also be very good candidates to interact with and then remove biofilms since they offer a large number of external surface groups controlling most of the physical properties and able to contemporaneously interact with active sites (Zarena and Gopal, 2013). Furthermore, they show complete solubility in water and can be charged on the surface to promote the interaction with the bacterial wall (Chen and Cooper, 2000; Tülü and Ertürk, 2012) or with biofilm molecules, interfering with chemical bonds present in the matrix. Dendrimers have also been functionalized with biocide functional groups able to interact with specific bacterial targets (Chen and Cooper, 2000; Lazniewska et al., 2012). For example, dendrimeric peptides inhibited *E. coli* viability in both planktonic and biofilm states in a dose-dependent manner at concentrations ranging from 5 to 40 μM (Hou et al., 2009). Johansson et al. (2008) also referred that multivalent fucosyl-peptide dendrimers caused a complete inhibition of biofilm formation (IC_50_ ∼10 μM) and dispersion of already established biofilms in *Pseudomonas aeruginosa*, without affecting bacterial growth.

Polyamino-phenolic ligands are polyfunctional molecules containing one or more phenol, biphenol, naphthol, cathecol, and other hydroxy-aryl groups connected to a polyamine skeleton with linear or macrocyclic topology (Ambrosi et al., 2008). Due to the acidity of the phenol moieties and to the basicity of the amine groups, these compounds are amphiprotic and in aqueous solution can be present in different protonation forms depending on the pH, most of them with amphi-ionic character. Polyamino-phenolic ligands are able to interact with several substrates as cations (including ammonium salts and metal ions), anions, and neutral species with strong hydrogen bond donors, like carbohydrates, urea derivatives, peptides and other highly polar biomolecules (Steed and Atwood, 2009).

Therefore, considering that dendrimers have already been shown acting as anti-microbial agents both in planktonic and biofilm states (Chen and Cooper, 2000; Tülü and Ertürk, 2012; Zarena and Gopal, 2013), they were selected in this study to experiment their action in the prevention and control of biofilms formed by *L. pneumophila*. Furthermore, previously fabricated polyamino-phenolic ligands (Ambrosi et al., 2008) were chosen considering that the potential role of amphiprotic molecules in the inhibition of biofilm is already well known (Banin et al., 2006; Chang et al., 2012).

## Materials and Methods

### Sampling and Isolation

Putative *L. pneumophila* strains were isolated from hot water of several accommodation facilities (hotels, residences and camping sites) in Pesaro–Urbino area (Italy) and verified as belonging to the genus *Legionella* according to Italian legislation (Guidelines for prevention and control of legionellosis, 2000).

*Aeromonas hydrophila*, *Pseudomonas aeruginosa*, *Klebsiella pneumonia*, *Escherichia coli* strains were isolated from the environment and identified as previously reported (Ottaviani et al., 2011; Sabatini et al., 2013; Boi et al., 2015).

### Molecular and Serological Identification

Isolated *Legionella* strains were cultured on Charcoal Yeast Extract (CYE) agar added with *Legionella* Buffered Charcoal Yeast Extract (BCYE) growth supplement (BCYE agar) (Liofilchem, Roseto degli Abruzzi, Italy). Genomic DNA was extracted using DNeasy Blood and Tissue Kit (Qiagen, Hilden, Germany), according to the manufacturer’s instructions. The strains were confirmed as *L. pneumophila* by a multiplex species-specific PCR assay (Qasem et al., 2008), using a slight modified protocol. The sequence of *Legionella* genus-specific primers (L5SL9 – L5SR93) and *L. pneumophila*-specific primers (LmipL920 – LmipR1548), the target gene and the expected size of amplified DNA were previously reported. The reaction mixture (45 μL + 5 μL of sample DNA) consisted of 0.15 μM of L5SL9 and L5SR93 primers, 0.5 μM of LmipL920 and LmipR1548 primers, 200 μM dNTPs, 1.5 mM MgCl_2_, 1X PCR Buffer, and 1.25 U Hot-Rescue DNA polymerase (all from Diatheva, Fano, Italy). Amplification of target DNA was performed in a MultiGene Gradient Termal Cycler (Labnet International, Edison, NJ, USA): initial denaturation at 95°C for 10 min followed by 35 cycles (1 min at 95°C, 1 min at 51°C, 1 min at 72°C) and final extension at 72°C for 10 min.

In order to distinguish *L. pneumophila* Sg 1 from serogroups 2–15, a serological test was performed using *Legionella* Latex Kit (Liofilchem, Roseto degli Abruzzi, Italy), a rapid agglutination test.

Stock cultures were stored at -80°C in Brain Heart Infusion Broth (Oxoid, Basingstoke, UK) containing glycerol at 20% (v/v) until they were used.

### Biofilm Assay

*Legionella pneumophila* strains were tested for their ability to form biofilm in different media: tap chlorinated water, filtered tap chlorinated water, spring water, filtered spring water and filtered *Legionella* Buffered Yeast Extract (BYE) broth, composed by 10 g/L Yeast Extract (Liofilchem, Roseto degli Abruzzi, Italy), 10 g/L ACES Buffer, 1 g/L alpha-ketoglutaric acid, 0.4 g/L L-cystein hydrochloride monohydrate, 0.25 g/L Iron (III) pyrophosphate hydrate (all from Sigma–Aldrich, St Louis, MO, USA) (before filtering, pH was adjusted to 6.9 with KOH). Water was used to set up a condition very similar to that in water systems. Two types of water were employed to highlight the different bacterial ability to produce biofilm in chlorinated and non-chlorinated water. Filtered water (0.2 μm pore size filter) was also used to avoid the interference of autochthonous microorganisms that could make the results hard to interpret and reproduce. BYE broth was used to verify if water could be a limitation for *Legionella* biofilm formation.

The biofilm formation assay was performed using a slight modification of a method reported by Baldassarri et al. (2001). Briefly, *Legionella* strains grown on BCYE agar were harvested using the waters and the broth described above. Single strain cultures were then centrifuged at 3000 rpm for 30 min and the pellet was resuspended in the same substrates to obtain an absorbance (600 nm) of 0.7 (9 × 10^8^ CFU/mL). The suspensions were inoculated in 96-well polystyrene plates (200 μL/well). After 3, 6, and 9 days of incubation at 37°C in wet atmosphere, planktonic cells were removed and wells were gently rinsed with sterile distilled water to eliminate non-adhering bacteria. Adhered bacteria were fixed at 80°C for 10 min. After staining at room temperature for 10 min with Hucker’s crystal violet, the dye was removed and the wells were washed two times. Hence, 200 μL 95% (v/v) ethanol were added in each well to dissolve Hucker’s crystal violet. Biofilm formation was quantified by measuring the absorbance of the solubilized dye at 570 nm, using a plate reader (Thermo Electron Corporation, Vantaa, Finland). Strains were identified as strong (OD > 0.240), weak (0.120 < OD < 0.240) and non- (OD < 0.120) biofilm producers. ATCC 35984, ATCC 35983, and ATCC 12228 *Staphylococcus epidermidis* strains were used as controls for strong, weak and non-biofilm producers (Zhang et al., 2003; Taj et al., 2012). The major biofilm producer strain was used for further analysis.

*Pseudomonas aeruginosa, A. hydrophila*, *E. coli*, and *K. pneumoniae* strains were simultaneously tested for their ability to form biofilm over 3 days in filtered tap chlorinated water at 37°C in wet atmosphere, each strain alone and in association with *L. pneumophila* major biofilm producer. Mono-species biofilms were formed as described for *Legionella* strains; two-species and five-species biofilms were prepared using the same amount of each species to reach the final concentration of 9 × 10^8^CFU/mL (200 μL/well).

### 16S rRNA Gene Sequencing

The higher biofilm producer strain of *L. pneumophila* was characterized by sequencing of the 16S rRNA gene. PCR amplification of the 16S rRNA gene was performed by adding 5 μL of the extracted DNA to 45 μL of the reaction mixture: 0.8 μM of 27F (DeLong, 1992) and 1492R (Lane, 1991) primers, 100 μM dNTPs, 1.5 mM MgCl_2_, 1X PCR Buffer, 0.075 U HotStart Taq DNA polymerase (all from Qiagen, Hilden, Germany). PCR was carried out on iCycler BioRad Thermal Cycler as decribed by Kahlisch et al. (2010). PCR products were purified by MinElute PCR Purification Kit (Qiagen, Hilden, Germany) and used as a template for sequencing with Big Dye Terminator Sequencing Kit (Applied Biosystems, Foster City, CA, USA), using the same primers as those used in the PCR. Sequencing PCR products were purified by DyeEx 2.0 Spin Kit (Qiagen, Hilden, Germany), following the manufacturer’s instructions, and sequenced on ABI Prism 3100 Genetic Analyser. Forward and reverse sequences were analyzed and assembled using Sequencher 5.2.4 (Gene Codes Corporation, Ann Arbor, MI, USA) and blasted against Ribosomal Database Project (Cole et al., 2013).

### Assessment of Bacterial Cell Viability in Multi-Species Biofilms

In order to assess cell viability of *L. pneumophila* in multi-species biofilms, a reverse-transcription quantitative real-time PCR (RTqPCR) assay was used to quantify transcriptionally active (viable) *Legionella* cells, as described by Andreozzi et al. (2014). Briefly, a standard calibration curve was built using appropriate dilutions (1 × 10^-5^ to 1 × 10^-8^ ng/μL) of a purified 5S rDNA amplicon obtained from a PCR reaction performed using extracted DNA of *L. pneumophila* grown on BCYE agar. PCR, RTqPCR and melt curve analysis were conducted as previously reported. Two-species and five-species biofilms were grown in filtered tap water for 3 days in 12-well plates containing 2 mL/well bacterial suspensions, with the same amount of each species to reach the final concentration of 9 × 10^8^CFU/mL. After incubation at 37°C, planktonic cells were removed and wells were washed. Adherent biofilm cells were harvested in sterile distilled water, obtaining a final OD_600_ of 1.0. RNA was extracted using Total RNA Purification Kit (Norgen Biotek, Thorold, ON, Canada), and retrotranscription of RNA in cDNA was carried out by QuantiTect Reverse Transcription Kit (Qiagen, Hilden, Germany), according to the manufacturer’s instructions. RTqPCR reactions containing undiluted and diluted (1:10, 1:100, 1:1000) cDNA from every mixed biofilm were run together to account for potential qPCR inhibition. Experiments were executed in triplicate for each reaction and repeated three times in order to assess repeatability and reproducibility of the standard curve. The coefficient of variation (CV) and the limit of detection (LOD) were determined. Transcriptionally active *Legionella* cell number/mL was calculated for every mixed biofilm as previously described.

The presence of *P. aeruginosa, A. hydrophila*, *E. coli*, and *K. pneumoniae* viable cells in mixed biofilms was detected by performing a Plate Count assay. Serial 10-fold dilutions of the same two-species and five-species biofilm samples used for RNA extraction were prepared in Phosphate Buffer Saline (PBS) and five 10 μL drops of each dilution were placed onto selective media: Cetrimide Agar, Mac Conkey Agar (both from Liofilchem, Roseto degli Abruzzi, Italy) and m-Aeromonas Selective Agar Base (Havelaar) (Biolife, Milano, Italy). CFU count was performed after incubation at 37°C for 24 h.

### Chemical Compounds

**FIGURE 1:**
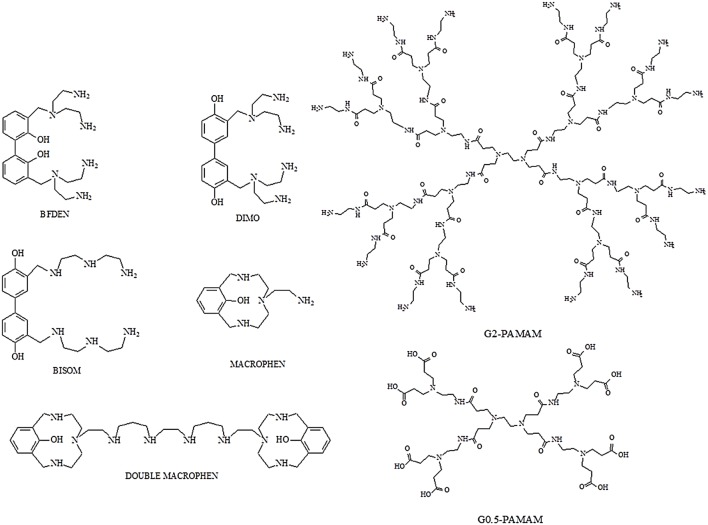
**Structures of polyamino-phenolic ligands and dendrimers.** BFDEN (3,3′-bis(N,N-bis(2-aminoethyl)aminomethyl)-2,2′-dihydroxybiphenyl), DIMO (3,3′-bis(N,N-bis(2-aminoethyl)aminomethyl)-4,4′-dihydroxybiphenyl), BISOM (3,3′-bis(N-(N-(2-aminoethyl)-2-aminoethyl)aminomethyl)-4,4′-dihydroxybiphenyl), MACROPHEN (2-(3,6,9-triaza-15-hydroxybicyclo(9.3.1)pentadeca-11,13,1^15^-trien-6-yl)ethylamine), DOUBLE MACROPHEN (1,16-bis(3,6,9-triaza-15-hydroxybicyclo(9.3.1)pentadeca-11,13,1^15^-trien-6-yl)-3,7,10,14-tetraazahexadecane), G0.5 PAMAM and G2 PAMAM.

Five polyamino-phenolic ligands (Macrophen, Double Macrophen, Bisom, Dimo, Bfden), synthesized by the Laboratory of Supramolecular Chemistry (Department of Pure and Applied Sciences, University of Urbino) (Ambrosi et al., 2008), and two PAMAM dendrimers half-generation (G0.5) and second-generation (G2), a gift from Prof. Donald A. Tomalia, were used to inhibit biofilm formation and promote biofilm degradation (**Figure 1**).

### Cytotoxicity Test

INT 407 (ATCC CCL-6) and HeLa (ATCC CCL-2) cell lines were used for cytotoxicity assays. 100 μL cell suspensions were seeded at numbers of 1 × 10^5^ cell/mL on 96-well plates that were incubated for 24 h at 37°C in 5% CO_2_. Then the chemicals were added to each well to obtain different final concentrations (0.01, 0.1, 0.5, 1.0, 5.0 mM). Chemical effect was evaluated after 24 and 48 h of incubation. Cell viability was assessed by 3-(4,5-dimethylthiazol-2-yl)-5-(3-carboxymethoxyphenyl)-2-(4-sulfophenyl)-2H-tetrazolium (MTS) assay using CellTiter 96 AQueous Non-radioactive Cell Proliferation Assay (Promega, Madison, WI, USA), adding 20 μl of the MTS/PMS (phenazine methosulfate) solution to each well. MTS is reduced by dehydrogenase enzymes of living cells into a soluble purple formazan product. After 2.5 h of incubation, absorbance of formazan, proportional to the number of viable cells, was measured at 492 nm by the plate reader previously mentioned. Cells without chemicals were used as positive control (PC). The percentage of treated cell viability was evaluated setting the control to 100%.

### Anti-Biofilm Activity of Chemicals

The activity of chemicals was tested on the biofilm formed by the highest *L. pneumophila* producer strain and on multi-species biofilms formed by different microorganism associations (*L. pneumophila*/*P. aeruginosa*, *L. pneumophila*/*A. hydrophila*, *L. pneumophila*/*E. coli*, *L. pneumophila*/*K. pneumoniae*, *L. pneumophila*/*P. aeruginosa*/*A. hydrophila*/*E. coli*/*K. pneumoniae*). These bacteria can exist in tap water and could interact with *Legionella* biofilm formation. Biofilm assay was carried out as described above.

#### Biofilm Degradation

Bacterial cultures in tap water (9 × 10^8^CFU/mL) were inoculated in 96-well polystyrene plates (200 μL/well); legionella mono-species and mixed biofilms were prepared as described above. The plates were incubated for 3 days at 37°C in wet atmosphere to allow biofilm formation. After incubation, planktonic cells were removed and wells were delicately rinsed with sterile distilled water to eliminate non-adhering bacteria. Then polyamino-phenolic ligands and PAMAM dendrimers were diluted in tap water (0.1 and 1.0 mM final concentration respectively) and added to the wells. The plates were incubated at 37°C for other 3 days to let the substances act on the already formed biofilms.

#### Biofilm Inhibition

Ten microliter/well polyamino-phenolic ligands (0.1 mM final concentration) and 10 μL/well PAMAM dendrimers (1 mM final concentration) were added to the 96-well polystyrene plates inoculated with bacterial cultures in tap water (9 × 10^8^CFU/mL) (190 μL/well) at the inoculum time to test biofilm inhibition activity of the chemicals over 3-day contact time.

Afterward, biofilm degradation and inhibition were evaluated by measuring the absorbance with the plate reader, as described above. Wells containing bacteria without chemical compounds were used as PC. Chemical activity was expressed as the percentage of biofilm reduction relative to the untreated control biofilm.

### Planktonic Microbial Cell Viability after Chemical Treatment

In order to determine if anti-biofilm activity of the chemicals was due to their microbicidal action or to direct interaction with the biofilm matrix, antimicrobial activity was determined on planktonic bacteria.

Microbial viability after compound treatments was tested on planktonic bacteria by flow cytometric analyses with a FACScalibur (Becton Dickinson, Milan, Italy) equipped with an Argon Ion Laser set at 15 mV and tuned to an excitation wavelength of 488 nm. Chemical microbicidal activity on free-floating microorganisms was evaluated: bacterial suspensions in PBS (9 × 10^8^CFU/mL) were treated with chemicals at the concentration used for biofilm experiments and incubated at 37°C for 24 h with agitation at 150 rpm, to avoid cell adhesion to flask bottom. Bacterial suspensions in PBS without chemicals were used as negative controls, while bacteria treated with 50 mg/L chlorine were used as PCs. To detect cell viability, bacterial cells were stained for 15 min with SYBR Green I (1:10000 final concentration) and Propidium Iodide (PI, 1 μg/mL final concentration) (both from Life Technologies, Waltham, MA, USA), fluorochromes with a high affinity for nucleic acids. Then cells were fixed in paraformaldehyde (4% final concentration) for 1 h, washed by centrifugation (3500 rpm for 15 min) and suspended in PBS. Cell count was performed by adding Cytocount^TM^ counting beads (DakoCytomation, Denmark) to the stained samples before processing by flow cytometry (Manti et al., 2011). Multi-parametric analyses were performed on both scattering signals (FSC, SSC), and FL1 to detect SYBR Green I fluorescence and FL3 to detect PI fluorescence. Three populations were evidenced in dot plots: viable cells, damaged cells and dead cells. All parameters were collected in logarithmic scale. All data were statistically analyzed with Cell Quest software.

### Scanning Electron Microscopy (SEM)

The successful biofilm formation by the major biofilm producer *Legionella* strain and the anti-biofilm activity of dendrimers were confirmed by electron microscopy. The strain grown on BCYE agar was harvested in filtered tap water as described above. One mililiter suspension adjusted at concentration of 9 × 10^8^ CFU/mL with or without dendrimers (1 mM) was inoculated in 24-well plate with coverslips. After 3 days of incubation at 37°C in wet atmosphere, non-adhering bacteria were removed by gently washing. Adhering bacteria growing on coverslips, were fixed with 2.5% glutaraldehyde in 0.1 M phosphate buffer (pH 7.3) for 1 h. The monolayer was washed and postfixed with 1% OsO_4_ in the same buffer for 1 h. A progressive alcohol dehydration was performed, followed by specimen critical point drying. After mounting on conventional SEM stubs by means of silver glue, slides were gold sputtered (Battistelli et al., 2005). Observations were carried out with a Philips 515 scanning electron microscope.

### Statistical Analysis

All experiments were executed in triplicate for each sample and repeated three times. Data were presented as means and standard deviations of the measurements. The reduction of biofilm was analyzed by two-way ANOVA model with respect to microorganisms and different compounds. Experiment wise significance level was fixed at *P* < 0.05. All the analyses were performed using Stata 12.1 SE (Stata corporation, College Station, TX, USA).

## Results

### Isolation and Identification of *Legionella* sp.

Ten putative *Legionella* strains were isolated from accommodation facilities in Pesaro–Urbino area (Italy) and confirmed as *Legionella* by biochemical tests. The genus/species-specific PCR assay reconfirmed the isolates belonged to *Legionella* genus and allowed to identify six *L. pneumophila* strains. *Legionella* Latex Kit revealed that all *L. pneumophila* strains were Sg 1.

### Microorganism Biofilm Producing Ability

Overall, *L. pneumophila* biofilm production assays showed that two strains were not able to form any biofilm (OD < 0.120), three of them were identified as weak biofilm producers and one strain (*L. pneumophila* 155) resulted a high biofilm producer (OD > 0.240). Based on the 16S rRNA sequence identity level, *L. pneumophila* strain 155 was classified as *L. pneumophila* subspecies *pneumophila* (GenBank accession number: KM657957) and as serogroup 1, after *Legionella*-specific serotyping assay.

Biofilm producer strains were able to form biofilms in all different growth substrates (data not shown). OD values showed that biofilm formation ability was not decreased when supplying water as growth medium instead of BYE broth. Biofilm formation in spring and tap chlorinated water was similar and not related to the substrate type: weak biofilm producer strains showed mean OD values of 0.144, 0.123, 0.186 in spring water and 0.120, 0.145, 0.172 in tap water for each strain, respectively; *L. pneumophila* 155 exhibited mean OD values of 0.265 in spring water and 0.281 in tap water. These data mean that chlorine concentration in tap water was not a limiting factor for biofilm formation. Bacteria were also able to form biofilm in filtered water as much as in unfiltered water (*P* > 0.05). Hence, filtered tap water was chosen as substratum for further experiments. Biofilm formation over 6 and 9 days did not show any increase in OD values compared with biofilms over 3 days, therefore 3-day biofilm was chosen for further experiments. *L. pneumophila* 155 was selected to study biofilm formation in association with the other environmental microorganisms.

**FIGURE 2:**
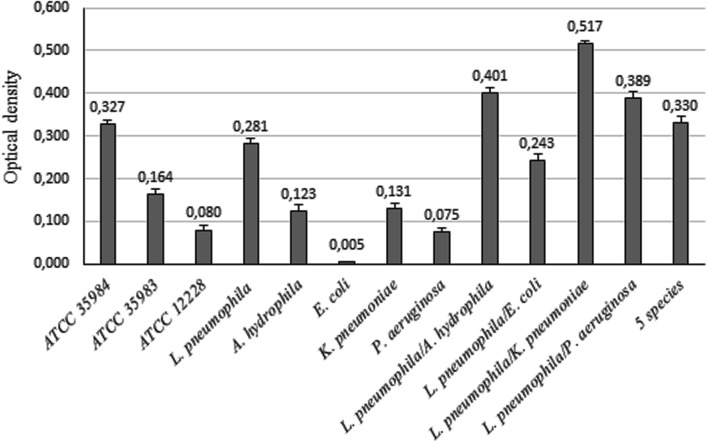
**Biofilm formation ability of bacterial strains.** OD values of biofilms formed by single bacterial strains and by *L. pneumophila* 155 in association with environmental strains. ATCC 35984, ATCC 35983, and ATCC 12228 *S. epidermidis* strains were used as controls for strong, weak and non-biofilm producers.

*Aeromonas hydrophila* and *K. pneumoniae* were classified as weak biofilm producers over 3 days in tap water (mean OD values of 0.123 and 0.131 respectively), whilst they strongly increased biofilm formation when in association with *L. pneumophila* 155. Instead *E. coli* and *P. aeruginosa* were not able to make biofilm on their own (OD < 0.120), but they enhanced the biofilm formation ability in presence of *L. pneumophila* 155 (**Figure 2**).

### Assessment of Bacterial Cell Viability in Multi-Species Biofilms

Reverse-transcription quantitative real-time PCR results demonstrated the presence of viable *L. pneumophila* 155 cells in both two-species and five-species biofilms. In particular, the highest viable cell number was found in *L. pneumophila* 155/*K. pneumoniae* biofilm (8.92 × 10^7^ cell/mL), followed by *L. pneumophila* 155/*A. hydrophila* biofilm (8.71 × 10^7^ cell/mL) (**Table 1**). CV intra- and inter-assay values (not higher than 8.5 and 8.7% respectively) proved the repeatability and the reproducibility of the assay. The LOD was 3.89 × 10^1^ copies of 5S rDNA/reaction, corresponding to 130 *L. pneumophila* 155 cells/mL. The RTqPCR assay displayed an overall efficiency of 91% and a Pearson’s R coefficient of 0.99. Melt curve analysis showed a clear and reproducible melting peak between 81.8 and 82.3°C.

**Table 1:** Count of viable *Legionella pneumophila* 155 cells and viable non-legionella cells in multi-species biofilms based on quantification of 5SrRNA copies by RTqPCR and on Plate Count assay, respectively.

Biofilms	Trascriptionally active *L. pneumophila* cells/mL	Non-legionella CFU/mL
*L. pneumophila*/*A. hydrophila*	8.71 × 10^7^ ± 6.92 × 10^6^	7.00 × 10^8^ ± 1.00 × 10^8^
*L. pneumophila*/*E. coli*	2.02 × 10^7^ ± 4.21 × 10^6^	5.67 × 10^8^ ± 1.53 × 10^8^
*L. pneumophila*/*K. pneumoniae*	8.92 × 10^7^ ± 6.30 × 10^6^	1.10 × 10^9^ ± 1.59 × 10^8^
*L. pneumophila*/*P. aeruginosa*	6.96 × 10^7^ ± 6.59 × 10^6^	2.11 × 10^8^ ± 6.49 × 10^7^
Five-species	4.98 × 10^7^ ± 3.20 × 10^6^	

*Cell number is expressed as mean ± SD.*

Plate Count assay showed the presence of viable non-legionella cells in all two-species biofilms (**Table 1**). However, it was not possible to quantify non-legionella viable strains in five-species biofilms because of the incomplete selectivity of the media, which allowed a widespread growth of *P. aeruginosa*.

### Chemical Cytotoxicity

Cytotoxicity assay was executed to determine the highest non-cytotoxic concentration that could be used against the microbial biofilm. Compound concentrations maintaining cell viability higher than 85% were chosen to test their anti-biofilm activity. A higher threshold than the standard value usually used to establish cytotoxic concentrations (IC_50_: half maximal inhibitory concentration), was used in this study.

**FIGURE 3:**
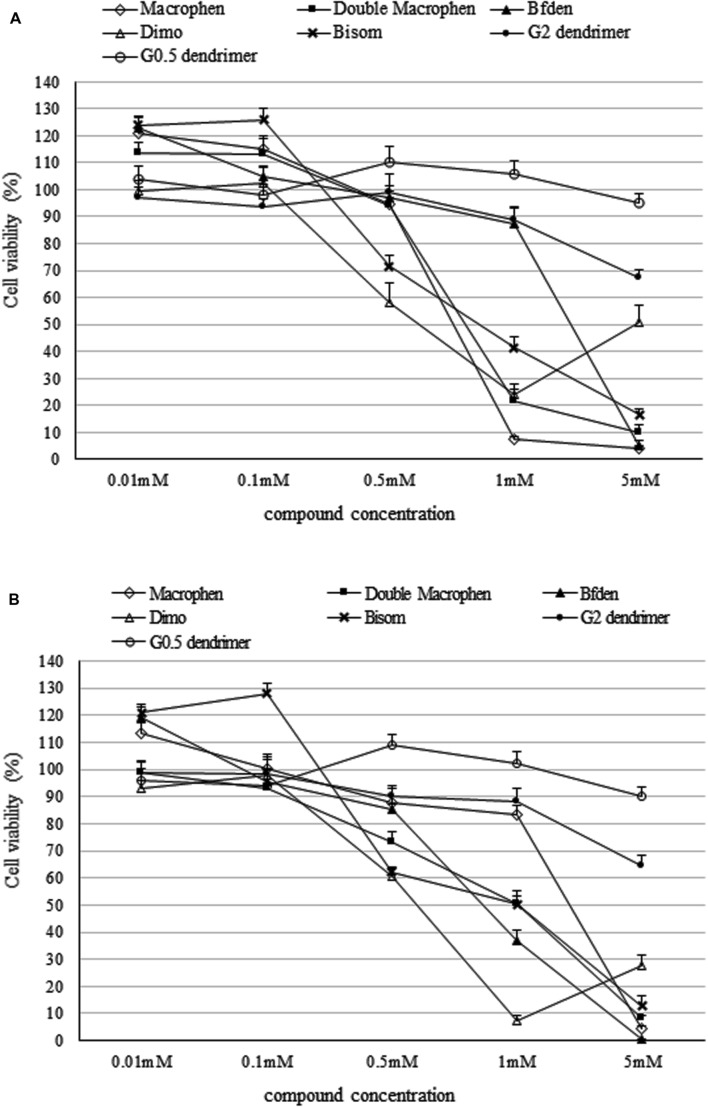
**Cytotoxicity of dendrimer and polyamino-phenolic ligands.** Cell viability percentage in INT 407 **(A)** and HeLa **(B)** cells after 48 h of treatment with dendrimers and polyamino-phenolic ligands at different concentrations. Untreated cells are set as 100%. Survival ratios are expressed as means ± SD.

**Figure 3** shows the toxicity of polyamino-phenolic ligands and dendrimers in INT 407 (**Figure 3A**) and HeLa (**Figure 3B**) cells respectively. Forty eight hour cell treatment exhibited a slightly higher cytotoxicity behavior compared with 24 h treatment, hence only 48 h data are illustrated below. Every compound showed nearly the same trend in both cell lines. When treated with polyamino-phenolic ligands, INT 407 and HeLa cells exhibited viability higher than 85% in the presence of all compounds at concentrations up to 0.1 mM. On the other hand, INT 407 cell viability was higher than 85% only after treatment with Macrophen, Double Macrophen or Bfden at 0.5 mM (94, 95, and 97% respectively). Treatment with 0.5 mM Bisom or Dimo led to a INT 407 cell viability of 72 and 58% respectively. Regarding HeLa cells, only Macrophen showed low cytotoxicity at the 0.5 mM concentration with a cell viability value of 88%, whereas cell viability was below the 85% threshold with the other compounds. G0.5 and G2 dendrimer treated cells maintained viability values higher than 85% up to 5 and 1 mM in both cell lines, respectively.

Therefore polyamino-phenolic ligands were used at 0.1 mM and dendrimers were used at 1 mM for further experiments on biofilms.

In addition, polyamino-phenolic ligand concentrations ≤0.1 mM and G0.5 dendrimer concentrations ≤0.5 mM increased MTS assay OD values of the cells up to 124%. The increased OD values at low chemical concentrations may reflect an increase of metabolic activity, due to cell-chemical interaction.

### Chemical Activity

**FIGURE 4:**
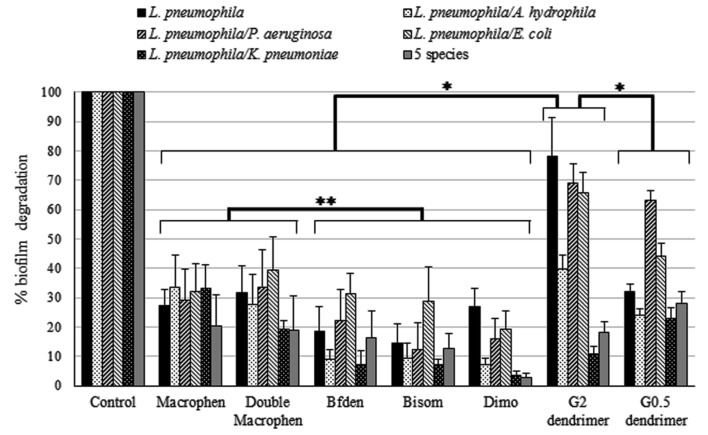
**Biofilm degradation activity of chemicals.** Degradation of legionella mono-species and multi-species 3-day-old biofilms by chemicals. Biofilms were treated with 0.1 mM polyamino-phenolic ligands and 1 mM dendrimers after 3-day biofilm formation. Untreated biofilms were acted as controls (100%). Degradation percentages are expressed as means ± SD. Asterisks indicate significant (^∗^*P* < 0.05) and non significant (^∗∗^*P* > 0.05) differences.

**FIGURE 5:**
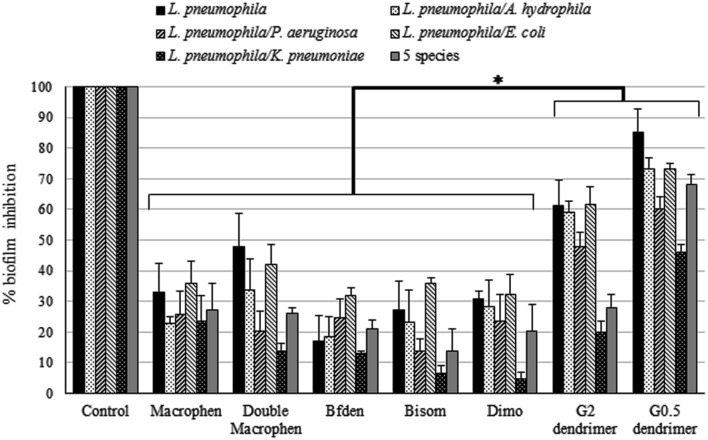
**Biofilm inhibition activity of chemicals.** Inhibition of legionella mono-species and multi-species biofilm formation by chemicals. Biofilms were treated with 0.1 mM polyamino-phenolic ligands and 1 mM dendrimers during 3-day biofilm formation period. Untreated biofilms were acted as controls (100%). Inhibition percentages are expressed as means ± SD. Asterisks indicate significant (^∗^*P* < 0.05) differences.

The percentages of biofilm reduction and inhibition by the chemical compounds are reported in **Figures 4** and **5**.

#### Biofilm Degradation

Dendrimers showed the highest biofilm reduction activity with both legionella mono-species and multi-species systems when compared to all other compounds (**Figure 4**). In general, G2 dendrimer was the most active in biofilm degradation, with statistically significant differences with relation to all compounds (*P* < 0.05), causing a reduction of 78% on *L. pneumophila* biofilm, while the highest decrease in multi-species biofilms was observed in *L. pneumophila*/*P. aeruginosa* (69%) and *L. pneumophila*/*E. coli* (66%). G0.5 dendrimer caused a similar reduction (63%) only on *L. pneumophila*/*P. aeruginosa* biofilm.

Among the polyamino-phenolic compounds, Macrophen and Double Macrophen were overall the most effective chemicals, but no statistically significant differences were found (*P* > 0.05). In particular, Double Macrophen showed the highest reduction on *L. pneumophila*/*E. coli* biofilm (39%), while Macrophen was the most effective on *L. pneumophila*/*A. hydrophila* and *L. pneumophila*/*K. pneumonia* (both 33%). Macrophen, Double Macrophen and Dimo were the most efficacious compounds against *L. pneumophila* biofilm (27, 32, and 27% respectively).

#### Biofilm Inhibition

When biofilm formation inhibition was evaluated (**Figure 5**), dendrimers showed the highest inhibition activity, with statistically significant differences in relation to all other compounds (*P* < 0.05). G0.5 dendrimer was, overall, the compound that showed the best efficacy, abundantly inhibiting legionella mono-species (85%) and *L. pneumophila*/*A. hydrophila* and *L. pneumophila*/*E. coli* (both 73%) two-species biofilms. G2 dendrimer also caused a rather high biofilm inhibition, although less effective, on the same biofilms as G0.5 (61, 59, 61% for *L. pneumophila*, *L. pneumophila*/*A. hydrophila* and *L. pneumophila*/*E. coli* biofilms respectively). Moreover, five-species biofilm was highly inhibited by G0.5 dendrimer, with a value of 68%, more than twice as much as the other compounds.

Among the polyamino-phenolic ligands, Double Macrophen was the most effective on *L. pneumophila* biofilm (48%). With respect to multi-species biofilms, Macrophen caused the highest inhibition on *L. pneumophila*/*P. aeruginosa* (26%), *L. pneumophila*/*K. pneumoniae* (24%) and five-species (27%) biofilm formation, whereas Double Macrophen was the main inhibitor of *L. pneumophila*/*A. hydrophila* (34%) and *L. pneumophila*/*E. coli* (42%) biofilms.

### Planktonic Microbial Cell Viability after Chemical Treatment

**FIGURE 6:**
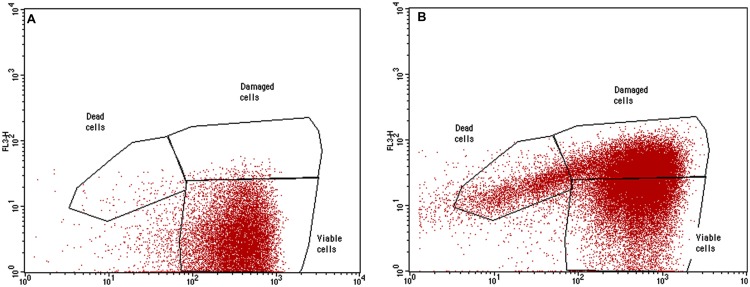
**Representative dot plot (FL1 vs. FL3).** Planktonic *E. coli* cells labeled with SYBR Green I and PI treated with G2 dendrimer **(B)** respect to the untreated control **(A)**.

**FIGURE 7:**
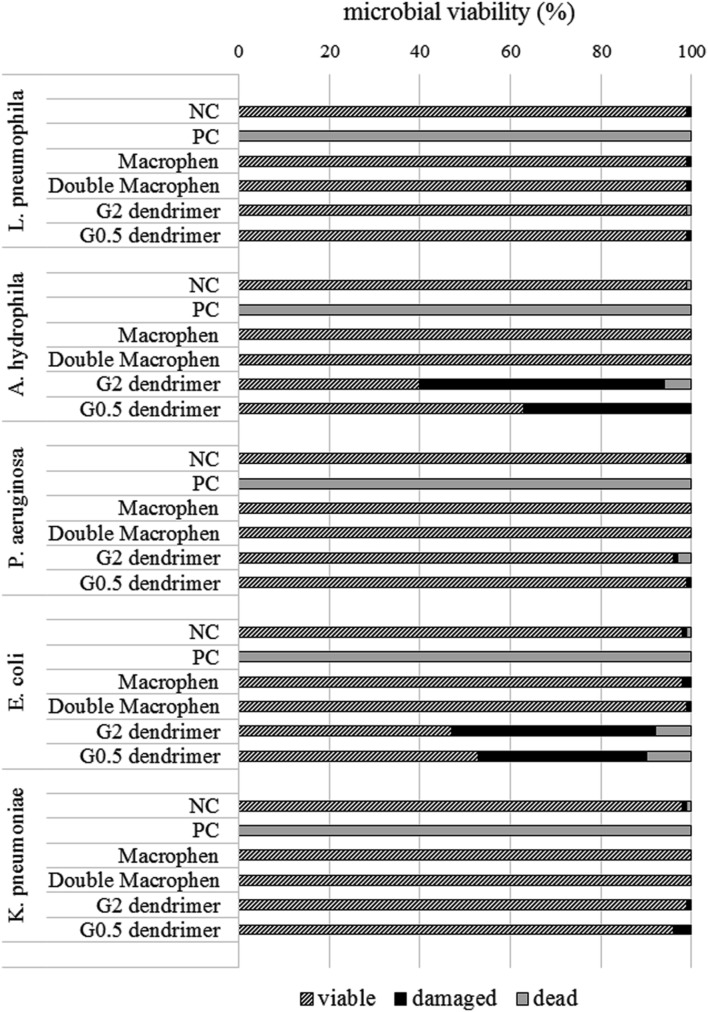
**Microbial viability.** Planktonic cell viability percentage obtained by flow cytometry analysis after 24 h chemical treatment with dendrimers (1 mM) and polyamino-phenolic ligands (0.1 mM). Untreated bacteria were used as negative controls (NC) (100%), while bacteria treated with chlorine (50 mg/L) were used as positive controls (PC).

The most active chemicals (G2 and G0.5 dendrimers, Macrophen and Double Macrophen) against biofilms were selected to determine their antimicrobial activity on planktonic bacteria by flow cytometry. Viability test showed that Macrophen and Double Macrophen treatments did not reduce the number of viable cells in all microbial species, while G2 and G0.5 dendrimer treatments reduced the viability only in *A. hydrophila* (60 and 37% respectively) and *E. coli* (53 and 47% respectively) (**Figure 6** shows a representative dot plot), with a parallel increase in damaged cell number (**Figure 7**).

### Scanning Electron Microscopy

**FIGURE 8:**
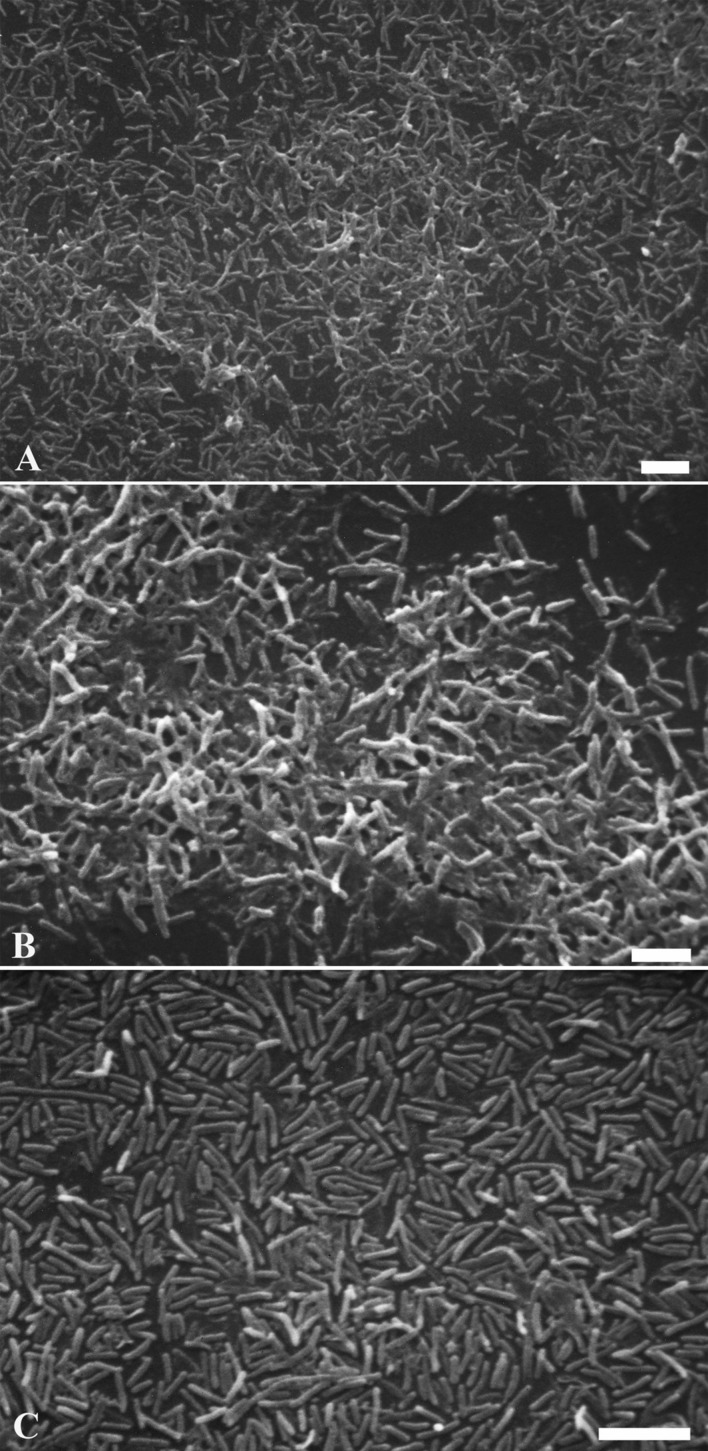
***Legionella pneumophila* 155 biofilm scanning electron micrographs.** Images showing biofilms formed by untreated *L. pneumophila* 155 cells **(A,B)** and *L. pneumophila* 155 + G2 dendrimer (1 mM) treatment **(C)**. An abundant formation of diffuse masses of biofilm with extracellular matrix is visible in **(A,B)**, while naked bacteria on the surface without extracellular matrix are shown in **(C)**. **(B)** is a higher magnification of **(A)**. **(A–C)** Bar = 5 μm.

The anti-biofilm activity of dendrimers was tested by SEM image analysis. After 3 days incubation, SEM revealed the formation of an abundant *L. pneumophila* 155 biofilm, extensively spread on the coverslip (1 cm diameter). **Figures 8A,B** (control sample) shows bacteria adhering to the substrate and embedded in an extracellular matrix, clearly visible on the surface of the aggregated cells. In the treated samples no extracellular matrix is visible: naked bacteria, mainly forming a monolayer, are attached to the surface, but there is no connection between cells (**Figure 8C**). The presence of dendrimers caused the lack of extracellular matrix, resulting in the detachment of a relevant cell amount that may justify the high reduction percentages obtained in the biofilm inhibition assay.

## Discussion

*Legionella pneumophila* biofilm studies are usually carried out in culture media and at different incubation temperatures (Piao et al., 2006). Some projects, however, require experimental conditions more similar to the environment where bacteria are normally found. Investigation into chemical action against microbial biofilms is more reliable when conducted using environmental-like conditions. Hence, in this study filtered tap water was chosen as substrate to better mimic water-network environment, where these chemicals are expected to perform their action. Biofilms were formed at 37°C to support legionella survival and reproduce hot water system temperature, according to Portier and Héchard (2013).

*Legionella pneumophila* 155 was shown to be a biofilm producer both in BYE medium and in water based on the OD value obtained by Hucker’s crystal violet assay. Moreover, demonstration of biofilm formation in water was confirmed by SEM, showing bacterial adhesion to surface and extracellular matrix connecting cells.

Pécastaings et al. (2010) demonstrated that *L. pneumophila* was able to form mono-species biofilm in low-nutrient medium not supporting planktonic growth and allowing bacterial replication only in the sessile phase, unlike classical rich media. Here, *Legionella* biofilm formation seemed not to be compromised in filtered water compared with culture media after 3-day incubation. Matrix presence allows us to refer to a biofilm in the initial formation phase, even though its production was probably due to planktonic cell deposition rather then to cell replication. In fact, matrix production could result from the high initial bacterial inoculum that might determine the activation of the Quorum Sensing and, consequently, biofilm formation. However, matrix exopolysaccharides could act as source of nutrients, supporting bacteria survival and possible growth.

In experimental conditions mono-species biofilms show a higher reproducibility than multi-species biofilms. On the other hand, multi-species biofilm assays are necessary to investigate the actual chemical power in a model closer to the environment where the compounds should be used.

The formation of mixed biofilms was shown using Hucker’s crystal violet assay, while the ability of viable bacteria to coexist were assessed by RTqPCR and CFU count assays. These methods were used only with the aim to verify the presence of viable bacteria; comparison between cell numbers of different bacterial species cannot be proposed because of the different quantification methods used (RTqPCR assay and CFU count) and the use of different selective media in CFU counts, that may vary in their ability to recover viable cells.

Interactions of different bacterial strains in mixed biofilms may turn into both synergy and reduction in biofilm production (Carpentier and Chassaing, 2004; Marouani-Gadri et al., 2009). Microbial interactions can determine appreciable biomass variation in two species biofilms in comparison with mono-species biofilms, and strains considered poor biofilm formers by themselves may be present in multi-species biofilms (Simões et al., 2007; Røder et al., 2015). Ren et al. (2015) also observed synergistic interactions with greater biofilm biomass production in multi-species communities compared with single-species cultures. Based on CV assay OD values, *K. pneumoniae* showed the highest synergistic effect (probably due to the positive interaction of the molecules involved in legionella and klebsiella Quorum Sensing mechanisms), proving to be the best species for *L. pneumophila* 155 to form biofilm with, followed by *A. hydrophila*. According to this result, Stewart et al. (2012) recently demonstrated that *L. pneumophila* is able to adhere and persist in *K. pneumoniae* dynamic biofilm. Considering bacteria that are not able to form biofilm, they could be able to adhere to an already formed biofilm or to other bacteria that have colonized a surface. In this study, *E. coli* and *P. aeruginosa* seem to be unable to form biofilm on their own, but biofilm formation happens in presence of *L. pneumophila*. They probably adhere to *L. pneumophila* cells, attached to the well surface, taking part in the biofilm biomass. *L. pneumophila/E. coli* biofilm OD is lower than *L. pneumophila* mono-species biofilm OD: in this case the presence of *E. coli* could slightly decrease *L. pneumophila* ability to produce biofilm. On the other hand *L. pneumophila/P. aeruginosa* biofilm OD is higher than *L. pneumophila* monospecies biofilm OD: it can be assumed that *P. aeruginosa* could enhance legionella ability to form biofilm.

In this work non-functionalized PAMAM dendrimers [strong antibacterial agents, less expensive and less tricky to be synthesized than the functionalized ones (Calabretta et al., 2007)] were used for the first time against *Legionella* biofilms. The efficacy of the chemicals not only to prevent bacteria from producing biofilms but also to reduce previously formed biofilms appeared to be dependent on the microorganism species and on the chemical properties. Overall, dendrimers were the molecules that generally reached the highest percentage of biofilm inhibition and reduction. It has to be pointed out that dendrimers were ten times more concentrated than polyamino-phenolic ligands, due to the cytotoxicity results, and this could be the reason for the dendrimer best performance. Although polyamino-phenolic ligands could exhibit an increased efficacy at higher concentrations, chemical cytotoxicity has to be taken into account because it is a relevant feature when dealing with substances that could be used in water systems.

PAMAM dendrimers have been previously shown to be highly toxic to some bacteria: Calabretta et al. (2007) showed that G5 amino-terminated PAMAM dendrimer killed 50% (EC_50_) of *P. aeruginosa* at concentrations of 1.5 μg/mL; Lopez et al. (2009) also reported that the Minimum Inhibitory Concentration (MIC) values against *P. aeruginosa* were 6.3 μg/mL for G3 and 12.5 μg/mL for G5 PAMAM dendrimer, while the MIC values against *Staphylococcus aureus* were 12.5 μg/mL for both dendrimers. In our study, flow cytometric data revealed that both G2 and G0.5 dendrimers were able to decrease planktonic *A. hydrophila* and *E. coli* microbial viability, probably interacting with and damaging the bacterial outer membrane, while they were not microbicidal on planktonic *L. pneumophila, P. aeruginosa*, and *K. pneumoniae*, at the used concentration. Results regarding *E. coli* and *K. pneumoniae* agree with those by other authors (Xue et al., 2013), reporting that *E. coli* Minimum Bactericidal Concentration (MBC) ranges from 12.5 and 25 μg/mL for G2 dendrimer. On the contrary, results regarding *P. aeruginosa* contrast with literature data: Calabretta et al. (2007) referred that the EC_50_ for G5 PAMAM was about 170-fold lower than the concentration used in our work, whereas Xue et al. (2013) showed that the MIC for G2 PAMAM was about 35-fold lower. The different effect of the dendrimers on the same bacterial species may be due to the different behavior of the single strains, but also to the different bacterial concentrations used in various studies. To the best of our knowledge, data concerning *L. pneumophila* and *A. hydrophila* has not been reported yet.

Non-functionalized PAMAM dendrimers act through non-specific mechanisms, unlike functionalized dendrimers, acting through specific molecule interactions (Calabretta et al., 2007; Wang et al., 2010; Xue et al., 2013). Cationic amine groups on full-generation dendrimer (such as PAMAM G2) surface may favor the adsorption onto negatively charged bacterial wall by electrostatic attraction, and change membrane permeability eventually resulting in cell death (Calabretta et al., 2007; Tülü and Ertürk, 2012; Xue et al., 2013); particularly, in gram-negative bacteria the polycation dendrimer may bind the polyanion lipopolysaccharide (replacing the stabilizing divalent ions, calcium and magnesium), may diffuse through the cell wall and destroy the inner cytoplasmic membrane. Also half-generation carboxyl terminated dendrimers (such as PAMAM G0.5) may alter the structure and permeability of the bacterial outer membrane acting as a polyanion chelating calcium and magnesium ions (which bind and stabilize the phosphate group in the lipid layer) and leading to cell wall disruption (Wang et al., 2010).

In this study, despite the lack of microbicidal activity in the majority of plancktonic bacteria, dendrimers were overall highly active against biofilms, suggesting that the anti-biofilm efficacy was not mainly due to the chemical microbicidal activity. Bacterial adhesion, the initial phase of biofilm formation, could be limited by dendrimer interaction with cell surface or with ions (that promote microbial adhesion), changing wall properties and avoiding microbial attachment to the substrate. However, the lack of microbicidal activity might suggest that biofilm inhibition by dendrimers was more likely to be due to chemical interaction with bonds and differently charged molecules in the matrix than with bacterial cells. This hypothesis was also confirmed by SEM images showing that *L. pneumophila* 155 biofilm treated with dendrimers was lacking in extracellular matrix; dendrimers could prevent the molecules produced by legionellae from binding to form the extracellular polymeric substances (EPS) of the matrix, weakening the adhesion of the cells to the surface and cell to cell interaction. Nevertheless, these hypotheses have to be confirmed by further analysis on dendrimer mechanisms of action.

Polyamino-phenolic ligands showed a lower anti-biofilm activity compared to dendrimer action at the concentrations used, even if they also caused biofilm inhibition and degradation, especially Macrophen and Double Macrophen on mono-species biofilms. Polyamino-phenolic ligands can act as chelating agents, forming complexes with cations, such as calcium and magnesium. Deficiency in these divalent ions might destabilize the biofilm matrix, usually constituted by proteins, extracellular DNA and especially polysaccharides (Branda et al., 2005), which normally bind a large amount of cations, whose lack may damage the biofilm structure and cause its detachment (Turakhia et al., 1983). Furthermore, the interaction with the extracellular matrix is more likely than the interaction with bacterial wall, because of the lack of Macrophen and Double Macrophen microbicidal activity. Our work shows for the first time the reduction efficiency of polyamino-phenolic ligands and G2 and G0.5 PAMAM dendrimers on *L. pneumophila* biofilms in the initial stage of their formation in filtered tap water. In particular, dendrimers were the most effective compounds, thanks to their lower cytotoxicity that allowed the use of a higher concentration. The anti-biofilm activity was not achieved by chemical antimicrobial properties, but probably by chemical interaction with the electrostatic bonds present in the biofilm that allowed the partial detachment.

These data suggest that the studied compounds, especially dendrimers, could be seen as potentially interesting molecules that might be studied for a possible rule in support of new efficacious anti-biofilm disinfection treatments, releasing bacteria, as *Legionella* sp., from protective biofilms, making microorganisms susceptible to disinfectant concentrations usually used in water systems. Nevertheless, further experiments are needed to study biofilm inhibition efficacy in presence of protozoa and at different chemical concentrations and incubation times to identify the time required to obtain the maximum biofilm reduction not only in static but also in dynamic flow conditions. Furthermore, the study of the interaction of these compounds with the biofilm is crucial to better understand the mechanism of action that allows them to inhibit bacterial attachment or promote matrix detachment.

## Author Contributions

EA and AP conceived and designed the experiments. EA, FB, and LS performed the experiments. EA analyzed the data. MO made the dendrimers available for usage, was involved in the writing of the chemical description and dispensed helpful advice for dendrimer usage. LG sinthetized polyamino-phenolic ligands, was involved in the writing of the chemical description and dispenced helpful advice for polyamino-phenolic ligand usage. AM supplied cytometry analysis tools and methods, was involved in sample cytometric analysis and in the writing of the cytometric section. MB supplied SEM analysis tools and methods and was involved in SEM image capture. EA wrote the paper. AP and FB were involved in the writing and review of the article. AP and FB managed founds. All authors have approved the final version of the article. All authors agree to be accountable for all aspects of the work in ensuring that questions related to the accuracy or integrity of any part of the work are appropriately investigated and resolved.

## Conflict of Interest Statement

The authors declare that the research was conducted in the absence of any commercial or financial relationships that could be construed as a potential conflict of interest.
